# SEOM clinical guideline in nasopharynx cancer (2017)

**DOI:** 10.1007/s12094-017-1777-0

**Published:** 2017-11-02

**Authors:** M. Pastor, A. Lopez Pousa, E. del Barco, P. Perez Segura, B. Gonzalez Astorga, B. Castelo, T. Bonfill, J. Martinez Trufero, J. Jose Grau, R. Mesia

**Affiliations:** 1 Servicio de Oncología Médica, Hospital La Fe de Valencia, Valencia, Spain; 2 Servicio de Oncología Médica - IIBSP, Hospital Sant Pau, Barcelona, Spain; 3 Servicio de Oncología Médica Complejo Asistencial Universitario de Salamanca, Salamanca, Spain; 4 Servicio de Oncología Médica Hospital Clínico San Carlos, Madrid, Spain; 5 Servicio de Oncología Médica Hospital Universitario San Cecilio, Granada, Spain; 6 Servicio de Oncología Médica Hospital Universitario La Paz, Madrid, Spain; 7 Servicio de Oncología Médica Corporació Sanitària Parc Taulí, Sabadell, Spain; 8 Servicio de Oncología Médica Hospital Universitario Miguel Servet, Saragossa, Spain; 9 Servicio de Oncología Médica, Hospital Clínic i Provincial de Barcelona, Barcelona, Spain; 10 Servicio de Oncología Médica, Institut Català d’Oncologia – Badalona, Barcelona, Spain

**Keywords:** Nasopharynx cancer, Treatment guidelines, Locally-advanced disease, Recurrent/metastatic disease

## Abstract

Nasopharyngeal carcinoma (NPC) is distinct from other cancers of the head and neck in biology, epidemiology, histology, natural history, and response to treatment. Radiation therapy is an essential component of curative-intent of non-disseminated disease and the association of chemotherapy improves the rates of survival. In the case of metastatic disease stages, treatment requires platinum/gemcitabine-based chemotherapy and patients may achieve a long survival time.

## Introduction

Nasopharyngeal carcinoma (NPC) is distinct from other cancers of the head and neck in biology, epidemiology, histology, natural history, and response to treatment. These differences justify a different approach.

NPC is an unusual tumor in our country. In Europe, in 2012 the rate of incidence was 0.4 cases/100,000/year (in Spain, 0.5 cases/100,000/year) [1]. The incidence of NPC is two to threefold higher in males compared with females. NPC displays a distinct racial and geographic distribution, which is reflective of its multifactorial etiology. In endemic populations, risk appears to be due to an interaction of several factors: Epstein-Barr virus (EBV) infection, environmental factors, such as the high intake of preserved foods and smoking, and genetic predisposition.

## Methodology

Methodology SEOM guidelines have been developed with the consensus of ten OC oncologists from the cooperative group Spanish Group for the Treatment of Head and Neck Tumors (TTCC) and SEOM. To assign a level and quality of evidence and a grade of recommendation to the different statements of this treatment guideline, the Infectious Diseases Society of America–US Public Health Service Grading System for Ranking Recommendations in Clinical Guidelines was used (Table 1). The final text has been reviewed and approved by all authors.

**Table 1:** Strength of recommendation and quality of evidence score

Category, grade	Definition
Strength of recommendation	
A	Good evidence to support a recommendation for use
B	Moderate evidence to support a recommendation for use
C	Poor evidence to support a recommendation
D	Moderate evidence to support a recommendation against use
E	Good evidence to support a recommendation against use
Quality of evidence	
I	Evidence from ≥ 1 properly randomized, controlled trial
II	Evidence from ≥ 1 well-designed clinical trial, without randomization; from cohort or case-controlled analytic studies (preferably from > 1 center); from multiple time series; or from dramatic results from uncontrolled experiments
III	Evidence from opinions of respected authorities, based on clinical experience, descriptive studies, or reports of expert committees

## Diagnosis

### Pathological diagnosis

A definitive diagnosis is made by endoscope-guided biopsy of the primary tumor. Incisional neck biopsy or nodal dissection should be avoided as this procedure will negatively impact in the subsequent treatment.

The pathological diagnosis of NPC should be made according to the World Health Organization (WHO) classification [2] (Table 2). Basaloid squamous cell carcinoma was added to the WHO classification in 2005; there are few reported cases but these have an aggressive clinical course and poor survival.

**Table 2:** Histological classification (WHO 2005)

Keratinizing squamous cell carcinoma (WHO type I)
Non-keratinizing carcinoma: this is subdivided into:
Differentiated type (WHO type II)
Undifferentiated type (WHO type III)
Basaloid squamous cell carcinoma

For nonkeratinizing or undifferentiated histology, consider testing for EBV in tumor and blood. Common means for detecting EBV in pathologic specimens include in situ hybridization for EBV-encoded RNA (EBER) or immunohistochemically staining for latent membrane protein (LMP) [IIA]. The EBV DNA load within the serum or plasma may be quantified using polymerase chain reaction (PCR) targeting genomic sequences of the EBV DNA such as BamHI-W, EBNA, or LMP; these tests vary in their sensitivity [3] [IIA].

## Diagnosis and staging

The study should include:

- A complete medical history and general physical examination.
- Full exploration of the head and neck area (including endoscopic examination).
- Pathological diagnosis:
  Multiple direct biopsies of the primary tumor.
  Fine-needle aspiration (FNA) or biopsy of cervical lymph nodes.
- General blood analyses.
- Imaging tests:
  Cranial-cervical computed tomography (CT) scan or magnetic resonance (MR) scan.
  Chest-abdomen-pelvis CT scan.
  Bone scan.
  Positron emission tomography-CT (PET-CT).

CT and MR can be complementary in this regard: CT is superior for the study of bony structures and for the presence of cervical lymph nodes, while the MR provides a better assessment of the primary tumor location and of intracranial structures and retropharyngeal spaces. PET scan may assist in the accurate planning of radiotherapy treatment (RT). PET-CT scan can replace the traditional work-up for detection of distant metastatic disease [4] [IIIA].

(f) Special pathologic studies:
  Consider EBV/DNA testing [IIIB].

In pathologic specimens include EBER or LMP [IA].

(g) Nutritional and dental status assessment [5, 6].

Staging TNM classification has been modified in the 8th edition, 2017. There are two changes in nasopharynx T classifications relating to anatomic markers rather than depth of invasion. The previous T4 criteria “masticator space” and “infratemporal fossa” were used as synonyms, but their anatomic descriptions differ, sowing confusion among clinicians. These terms will now be replaced by a specific description of soft-tissue involvement to avoid ambiguity. In addition, adjacent muscle involvement (including medial pterygoid, lateral pterygoid, and prevertebral muscles) will now be “down-staged” to T2 based on a recent analysis showing them to have a more favorable outcome using current treatment [7].

In the N classification of nasopharynx, the iconic, traditional description of the supraclavicular fossa that was unique to this site will be replaced by contemporary definitions used for other head and neck sites and more suited to axial cross-sectional imaging. In addition, low neck involvement and > 6 cm size will be merged into a single N3 designation (formerly N3a and N3b), and T4 and N3 will both designate stage IVA (formerly IVA and IVB) in stage grouping (Tables 3 and 4).

**Table 3:** TNM staging classification (AJCC Cancer Staging Manual, 8th; 2017)

Primary tumor (T)
TX primary tumor cannot be assessed
T0 no tumor identified, but EBV-positive cervical node(s) involvement
T1s carcinoma in situ
T1: tumor confined to the nasopharynx, or tumor extends to oropharynx and/or nasal cavity without parapharyngeal extension
T2: tumor with extension to parapharyngeal space and/or infiltration of the medial pterygoid, lateral pterygoid, and/or prevertebral muscles
T3: tumor invades bony structures of skull base cervical vertebra, pterygoid structures, and/or paranasal sinuses
T4: tumor with intracranial extension and/or involvement of cranial nerves, hypopharynx, orbit, parotid gland and/or infiltration beyond the lateral surface of the lateral pterygoid muscle
Regional lymph nodes (N)
NX: regional lymph nodes cannot be assessed
N0: no regional lymph nodes metastasis
N1: unilateral metastasis, in cervical lymph node(s), and/or unilateral, or bilateral metastasis in retropharyngeal lymph nodes, 6 cm or less, above the caudal border of cricoid cartilage
N2: bilateral metastasis in cervical lymph node(s), 6 cm or less above the caudal border of cricoid cartilage
N3: metastasis in cervical lymph node(s) greater than 6 cm in dimension and/or extension below the caudal border of cricoid cartilage
Distant metastasis (M)
M0: no distant metastasis
M1: distant metastasis

**Table 4:** Stage grouping

Stage 0	T1s	N0	M0
Stage I	T1	N0	M0
Stage II	T1	N0, N1	M0
	T2	N0, N1	
Stage III	T0, T1, T2	N2	M0
	T3	N0, N1, N2	
Stage IVA	T4	N0, N1, N2	M0
	Any T	N3	
Stage IVB	Any T	Any N	M1

## Treatment

Radiation therapy (RT) is the mainstay of treatment and is an essential component of curative-intent treatment of non-disseminated NPC. Surgery has no role in the initial treatment given its particularities of the anatomy of the area. The role of surgery is limited at the moment to the salvage of residual disease or relapse [IA].

The techniques of 3D planning and intensity-modulated radiation therapy (IMRT) can improve outcomes without worsening toxicity and can offer a better protection of the different organs in the area that usually limit the dose of radiation that can be given (the use of IMRT can reduce the xerostomy frequently seen with the irradiation of the salivary glands) [IIA].

## Treatment of early stages (I and II)

The treatment for early stage tumors is RT, including both sides of the neck and retropharyngeal nodes. The dose should be 66–70 Gy to the primary tumor and affected lymph nodes areas, and 50 Gy to the uninvolved neck. In patients treated with IMRT alone, 5-year distant-metastases-free survival rate is 92–94% [8] [IA].

Given the significant toxicities of concurrent chemoradiotherapy (CT/RT) and the generally excellent prognosis of stage II nasopharyngeal cancer with IMRT, the role of administering chemotherapy (CT) concurrently with radiation in all stage II patients remains to be clearly defined, although consideration on individual bases should be made based on risk factors such as significant nodal disease, parapharyngeal tumor extension, and plasma EBV level [9] [IIB].

## Treatment of locally advanced stage (III and IV A/B)

Concurrent CT/RT is the standard treatment for locoregionally advanced nasopharyngeal carcinoma (with CDDP at 100 mg/m2 every 21 days) substantially improved locoregional control compared with exclusive RT, but distant metastasis is the main source of treatment failure [10] [IA].

Additional cycles of CT (with induction or adjuvant chemotherapy) could improve results and increases failure-free survival, overall survival, and distant failure-free survival with acceptable toxicity profile but its role is uncertain [IB].

A high rate of toxicity that usually leads to a low percentage of patients that are able to complete the adjuvant treatment and compliance is a significant problem with only about 50–75% of patients who were initially planned for adjuvant chemotherapy receiving the three planned cycles. Induction CT could avoid this problem [11, 12] [IIA].

The use of one or another should be tailored according to the patient’s clinical condition (ex, CT induction in highly symptomatic patients, adjuvant therapy to the rest). In patients with good general condition, TPF induction CT should be an option to be considered problem [9, 13, 14] [IIB].

When there is persistent cervical disease after standard CT/RT treatment, cervical rescue surgery should be performed. In cases with large cervical disease (N3), irrespective of the response to CT/RT, its systematic use could be considered. This could be especially relevant in cases with WHO type 1 histology WHO. However, the morbidity of this approach can be substantial and it has not been generally accepted. There are no studies to clarify this point definitively [IIIB].

## Recurrent and metastatic disease treatment (RM-NPSCC) (IV C)

In the setting of local and/or regional relapse, the multidisciplinary team should assess the possibility of salvage local therapy, whether by surgery or re-irradiation, with or without CT. These approaches can rescue a small percentage of cases, albeit at the cost of high toxicity. The election of one or another approach has not been well established [15]. The best results have been achieved when the previous interval free of disease is longer. If loco-regional relapse of NPSCC occurs, local treatment with surgery and/or chemo-radiotherapy is recommended [IIB].

When salvage treatment is not feasible or the patient develops a metastatic disease, the treatment of choice is palliative CT. A wide range of chemotherapy drugs has been tested mainly in retrospective and small phase II trials such as: platinum compounds (cisplatin, carboplatin), fluoropirimidines (5-fluorouracil, capecitabine), taxanes (paclitaxel, docetaxel), gemcitabine, anthracyclines, irinotecan and vinorelbine. Traditionally, the most used schedules included platinum-based combinations, mainly with 5-FU, with responses rates between 50 and 70% in retrospective uncontrolled studies [16–18].

A recent phase III randomized trial comparing cisplatin-5-FU with cisplatin-gemcitabine in 362 patients, showed a significant advantage in terms of progression-free survival in the gemcitabine-based cohort. Owing to no other phase III trials in this setting, this schedule has become the new standard first line approach in RM-NPC [19]. Cisplatin-gemcitabine is the first choice as first line palliative CT treatment in RM-NPSCC [IA].

To date, there is not an established standard treatment after the failure of the first line. If the patient has a good performance status, any of the previously reported active drugs could be considered but the inclusion in clinical trials should be encouraged.

## Follow-up (Table 5)

Evaluation of response in the nasopharynx and neck should be performed through clinical, endoscopic examination and imaging studies (18FDG-PET/CT scan or MR). A final assessment of the disease is recommended to be undertaken at 3 months after the end of the treatment to confirm the complete remission. MR is often preferred to evaluate the response to RT or chemo-radiotherapy, especially for T3 and T4 tumors, though distinction between post-irradiation changes and recurrent tumors may be difficult.

**Table 5:** Follow-up of nasopharyngeal carcinoma

Final assessment (2–3 months after the end of treatment)
Local and regional exam plus nasopharyngeal fibroscopy
FDG-PET/CT and/or RMI
First two years
Local and regional exam plus nasopharyngeal fibroscopy (every 3 to 4 months)
Chest X-ray, thyroid function test, CT/MRI (yearly)
Two to five years
Local and regional exam plus nasopharyngeal fibroscopy (every 6 months)
Chest X-ray, thyroid function test, CT/MRI (yearly)

Sometimes early detection of possible relapses could be managed with salvage treatment. Follow-up of the patients include periodic examination of the nasopharynx, neck, cranial nerve function and evaluation of systemic complaints to identify distant metastasis. Clinical exam and fibroscopy every three to 4 months during the first 2 years, then every 6 months until 5 years, then yearly should be followed. For T3 and T4 tumors, MR might be used on a 6- to 12-month basis to evaluate the nasopharynx and the base of the skull at least for the first few years after treatment. Thyroid function tests (if neck irradiated) and thoracic imaging test should be carried out at least once a year [3, 20].
